# The rs12526453 Polymorphism in an Intron of the *PHACTR1* Gene and Its Association with 5-Year Mortality of Patients with Myocardial Infarction

**DOI:** 10.1371/journal.pone.0129820

**Published:** 2015-06-18

**Authors:** Anna Szpakowicz, Marek Kiliszek, Witold Pepinski, Ewa Waszkiewicz, Maria Franaszczyk, Malgorzata Skawronska, Rafal Ploski, Anna Niemcunowicz-Janica, Beata Burzynska, Dorota Tulacz, Agata Maciejak, Marcin Jakub Kaminski, Grzegorz Opolski, Wlodzimierz Jerzy Musial, Karol Adam Kaminski

**Affiliations:** 1 Department of Cardiology, Medical University of Bialystok, Bialystok, Poland; 2 1st Chair and Department of Cardiology, Medical University of Warsaw, Warsaw, Poland; 3 Department of Cardiology and Internal Diseases, Military Institute of Medicine, Warsaw, Poland; 4 Department of Forensic Medicine, Medical University of Bialystok, Bialystok, Poland; 5 Laboratory of Molecular Biology, The Cardinal Stefan Wyszynski Institute of Cardiology, Warsaw, Poland; 6 Department of Medical Genetics, Medical University of Warsaw, Warsaw, Poland; 7 Institute of Biochemistry and Biophysics, Polish Academy of Sciences, Warsaw, Poland; University Hospital Medical Centre, GERMANY

## Abstract

**Objective:**

The rs12526453 (C/G) is a single nucleotide polymorphism in an intron of the *PHACTR1* gene (phosphatase and actin regulator 1). The C allele is associated with increased risk of coronary artery disease in an unknown mechanism. We investigated its association with long-term overall mortality in patients with ST-elevation myocardial infarction (STEMI) treated invasively.

**Methods:**

Two independent groups of patients with STEMI were analyzed: a derivation group (n= 638) and a validation one (n=348). Genotyping was performed with the TaqMan method. The analyzed end-point was total long term mortality. Additionally, transcriptomic analysis was performed in mononuclear blood leukocytes from rs12526453 CC monozygotes or G allele carriers.

**Results:**

In the study group (mean age 62.3 ± 11.9 years; 24.9% of females, n=159), percentages of CC, CG, and GG genotypes were 45.3% (n=289), 44.7% (n=285), and 10% (n=64), respectively. In the 5-year follow-up 105 patients died (16.46%). CC homozygotes had significantly lower mortality compared to other genotypes: 13.1% (n=38) vs. 18.3% in G-allele carriers (n=67), (p=0.017, Cox`s F test). In the validation group 47 patients died within 3 years (13.5%). We confirmed lower mortality of CC homozygotes: 10.1 % (n=18) vs. 16.95% in G-allele carriers (n=29), (p=0.031, Cox`s F test). Transcriptomic analysis revealed a markedly higher expression of NLRP-2 in CC homozygotes.

**Conclusions:**

The rs12526453 CC homozygotes (previously associated with increased risk of myocardial infarction) showed, in 2 independent samples, better long-term survival. The finding of such high effect size, after appropriate validation, could potentially be translated into clinical practice.

## Introduction

The rs12526453 is a single nucleotide polymorphism (SNP) of the 4^th^ intron in the phosphatase actin regulator 1 gene (*PHACTR1*, 6p24.1 locus) that has been related to early-onset myocardial infarction in a genome-wide association study (GWAS) performed in a population of the Myocardial Infarction Genetics Consortium [1]. This finding was replicated for association with various forms of coronary artery disease [2, 3] and ischemic stroke [4]. It has also shown significant association with coronary artery calcification, which is a major risk factor for coronary artery disease [5].

Another widely investigated SNP in the *PHACTR1* gene is rs9349379, also located in a non-coding region [6, 7]. Several further studies have confirmed its association with coronary artery disease [3, 8–10] and coronary artery calcification [7, 11]. Due to strong linkage disequilibrium with rs12526453 SNP on the haplotype level, the associations of rs9349379 may be extended to rs12526453.

PHACTR1 is a molecule expressed in the brain, lung, kidney, testis, and heart [12]. It inhibits protein phosphatase-1 (PP1) and binds actin via C-terminal domain [13]. It was recently shown to be involved in regulation of human umbilical vein endothelial cells’ apoptosis and angiogenesis [13]. Its biological effect and link to cardiovascular diseases, however, are still to be elucidated. Moreover, the effects described here of SNP on *PHACTR1* expression and regulation are unknown.

There are numerous GWASs that show an association between specific genomic regions and cardiovascular diseases, including myocardial infarction. Data concerning their influences on long-term prognosis and disease progression are still limited. Such studies consume much time and effort. So far, there are also no reports on the relation between *PHACTR1* polymorphisms and mortality after acute coronary syndromes. In general, one is allowed to expect the potentially adverse effect of a high-risk genotype on prognosis, but such a hypothesis needs confirmation. It is still unknown how these SNPs affect phenotype, especially in patients after acute coronary syndromes. Genotyping is one of the methods that might potentially improve risk stratification in this population. Risk assessment of patients after myocardial infarction that is based on conventional variables like ejection fraction, comorbidities, age, or risk scores (TIMI or GRACE), improves healthcare and points out who could benefit from the special attention of a physician. Enrichment of classical risk factors by new genetic ones may in future improve the prognosis of patients with STEMI.

The aim of the study was to investigate the association of the rs12526453 SNP of the *PHACTR1* gene with the long-term overall mortality in patients with ST-elevation myocardial infarction (STEMI) treated invasively. This is the first report on such an association.

## Materials and Methods

### Clinical assessment

We enrolled in the study consecutive patients with STEMI who were treated invasively and survived the first 48 hours after hospital admission. All patients were of European descent. The derivation group included subjects from Northeast Poland hospitalized in the years 2001–2005. The validation group was based on the population from central Poland who had STEMI in the years 2008–2010. Both sets came from tertiary reference centers and included patients transferred from local hospitals without invasive treatment facilities. Early-deceased patients were excluded, because the study aimed to investigate long-term mortality and the potential feasibility of genetic risk stratification. No further selection criteria were applied. In all cases coronary angiography was performed within 12 hours of symptoms onset (there were no patients with indications to angiography beyond 12 hours). Additionally, the control group included 161 subjects genotypically representative of our region (men and women who took part in paternity testing).

STEMI was diagnosed based on contemporary guidelines. The derivation set followed a definition of the World Heart Organization that included typical chest pain history, persistent ST-elevation in ECG (or potentially new left bundle branch block), and a typical pattern (a rise above the norm or a rise and fall) of cardiac necrosis markers (Troponin I or creatine kinase-MB in serial measurements) [14]. In the validation group the Universal Definition of Myocardial Infarction was applied [15].

Clinical characteristics included patients’ history, a physical examination performed on admission, routine laboratory tests, cardiac ultrasound, and a number of vessels with significant stenosis and the result of an invasive procedure. Estimated glomerular filtration rate (eGFR) was assessed with MDRD formula (Modification of Diet in Renal Disease) [16]. All patients also had the risk of death evaluated, based on clinical parameters included in the GRACE risk score [17]. Pharmacological treatment was based on contemporary guidelines.

The analyzed end-point was long-term all-cause mortality (5-year in the derivation group and 3-year in the validation group). Data on survival was retrieved from local population registries run by a Government Office, as described previously [18].

### Laboratory methods

EDTA blood samples were used for DNA extraction (Blood Mini kit, A&A Biotechnology). SNP was assessed with a TaqMan SNP Genotyping Assay on the ABI 7500 real time PCR platform (Applied Biosystems), according to manufacturer’s instructions. Every tenth sample was genotyped twice (quality control requirements).

#### Transcriptomic analysis

After uncovering the difference in survival between CC homozygotes and G-allele carriers, microarray and principal component analysis (PCA) were performed to investigate the potential effect of CC homozygosity on gene expression in the setting of STEMI. Forty-six subjects with STEMI were selected (23 in each group), matched for age. Their clinical characteristics are presented in S1 Table. Sodium-heparinized blood was collected at two time points: 0–24 hours from admission, and after 4–6 days (at discharge). The procedures for isolating peripheral blood mononuclear cells (PBMC), purification, RNA isolation, and GeneChip HUGene 1.0 ST Arrays (Affymetrix, Santa Clara, CA, USA) analysis were performed as described previously [19]. In the case of gene *NLRP2*, which was expressed differentially at both time points, microarray results were confirmed using quantitative RT-PCR. Detailed description of the procedure is presented in the S1 File.

### Statistical methods

Distribution of the continuous variables was determined with Shapiro-Wilk test. Clinical parameters of CC homozygotes and G-allele carriers were compared with Student`s t-test, Mann-Whitney or Chi^2^ tests, as appropriate. For survival analysis, we used Kaplan-Meier method with Cox`s F test. Univariate and multivariate analyses were performed with logistic regression. A primary model of multivariate regression included variables with significant association. The final model was selected in a backward stepwise manner. Microarray analysis was performed as described previously (See S1 File) [19]. Genes expressed differentially between groups were determined using the 1.3 fold cutoff.

Determination of sample size was performed with chi-square test. The study was designed to have a statistical power of at least 80 percent to detect a 70 percent relative risk increase in 5-year mortality of CC high-risk homozygotes compared to other genotypes. Predicted 5-year mortality rate was 20% [18] and the prevalence of CC homozygotes around 50% [2, 5]. Under those conditions the target of events would be achieved in a group of 608 patients.

The protocol of the study was approved by the Medical University of Bialystok Bioethics Committee and the Medical University of Warsaw Bioethics Committee. The study was performed in accordance with the ethical standards laid down in the 1964 Declaration of Helsinki. Informed written consent was obtained from all participants.

## Results

### Characteristics of the derivation cohort

We included in the derivation cohort 652 patients. 9 of them were lost to follow-up (1.4%) and in 5 cases genotype could not be determined due to poor sample quality (<1%). In samples genotyped twice, identical results were obtained. The final derivation group comprised 638 patients: mean age 62.3 ± 11.9 years, 24.9% of females (n = 159), TIMI 3 after PCI was obtained in 92.1% of patients (n = 588).

The percentages of CC, CG, and GG genotypes were 45.3 (n = 289), 44.7 (n = 285) and 10% (n = 64), respectively. In the control group (n = 161) it was 47.2% (n = 76), 41% (n = 66) and 11.8% (n = 19), consecutively (p = 0.64 compared to derivation group, Chi^2^ test). No significant deviations from Hardy-Weinberg equilibrium was found in either group (p = 0.6 and p = 0.45, consecutively). The specific allele frequencies are comparable to previous reports [4,7]. Clinical characteristics of CC homozygotes and G-allele carriers was shown in Table 1. CC homozygotes had a significantly lower heart rate on admission compared to other genotypes. No further significant clinical differences were found.

**Table 1:** Baseline characteristics of the study and validation groups.

Characteristic	Derivation group N = 638	CC homozygotes N = 289	G-allele carriers N = 349	P*	Validation group N = 348	P^
Age (years)	62.3 (11.9)	62.3 (12.2)	62.4 (11.6)	0.98	64.0 (11.5)	0.065
Female gender (%)	24.9 (n = 159)	23.9 (n = 69)	25.8 (n = 90)	0.57	28.5 (n = 99)	0.22
Hypertension (%)	54.5 (n = 348)	52.6 (n = 152)	56.1 (n = 196)	0.36	64 (n = 223)	0.038
Type 2 diabetes (%)	22.1 (n = 141)	20.4 (n = 59)	23.5 (n = 82)	0.35	21.3 (n = 74)	0.76
Smoking (%)^#^	48.3 (n = 219)	49.3 (n = 105)	47.5 (n = 114)	0.7	43.1 (150)	0.14
Previous myocardial infarction (%)	11.3 (n = 72)	10.7 (n = 31)	11.7 (n = 41)	0.68	13.8 (n = 48)	0.25
Systolic blood pressure (mmHg)	138.5 (28.2)	136.85 (26.4)	139.9 (29.6)	0.22	125.9 (21.5)	<0.0001
Heart rate (beats/min)	75.8 (17.8)	74.2 (18.1)	77 (17.4)	0.008	79.8 (17.2)	0.0005
Killip class III or IV (%)	6.6 (n = 42)	7.6 (n = 22)	5.7 (n = 20)	0.34	3.7 (n = 13)	0.063
Body mass index (kg/m2)	24.7 (3.7)	24.5 (3.7)	24.8 (3.7)	0.37	NA	NA
ST-elevation in anterior leads	39.2 (n = 250)	42.2 (n = 122)	36.7 (n = 128)	0.15	44 (n = 153)	0.14
TIMI flow grade 3 after procedure	92.1 (n = 588)	93.8 (n = 271)	91 (n = 317)	0.17	94 (n = 327)	0.29
Stent implantation (%)	77.1 (n = 492)	79.2 (n = 229)	75.4 (n = 263)	0.24	95.1 (n = 331)	<0.0001
Creatinine (mg/dl)	1.04 (0.4)	1.06 (0.5)	1.02 (0.3)	0.48	1.07 (0.6)	0.65
Ejection fraction (%)	45.8 (9.5)	45.3 (9.2)	46.1 (9.6)	0.17	45.2 (9.0)	0.28
Grace risk score	149.9 (35)	150.2 (35.5)	149.7 (35.3)	0.69	151 (32)	0.21

Mean values with standard deviations are given, unless otherwise specified.

* CC homozygotes vs. G-allele carriers.

^^^ derivation vs. validation group.

^#^Smoking status in the derivation group was available in 453 patients (in 213 CC homozygotes and 240 G-allele carriers). It was available in all patients from the validation group.

NA- data not available.

### Survival analysis- derivation cohort

**Fig 1:**
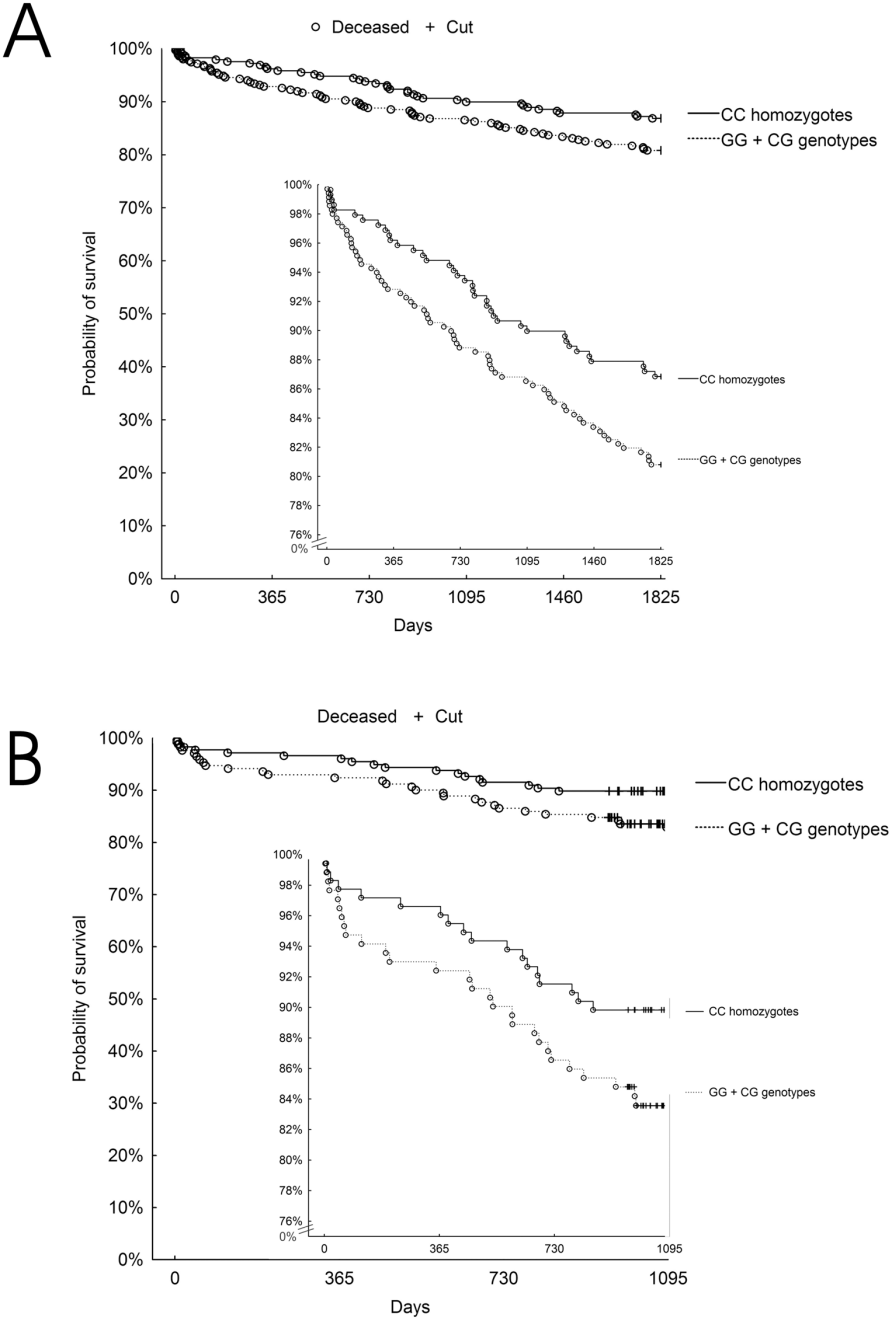
Kaplan-Meier survival curves for CC homozygotes and G-allele carriers of the rs12526453 SNP in the derivation group (panel A) and in the validation group (panel B). The differences between the groups were statistically significant (p = 0.017 in the derivation group and p = 0.031 in the validation group, Cox`s F test).

According to survival, at the cut-off point of 1825 days (5 years) 105 patients had died (16.46%). CC homozygotes (a genotype of increased risk of myocardial infarction) had significantly better survival compared to G-allele carriers (p = 0.017, Cox`s F test). 13.1% of CC homozygotes (n = 38) and 18.3% of G-allele carriers (n = 67), (p = 0.047, chi-2 test) died. Fig 1A presents Kaplan-Meier survival curves for both groups during the 5-year follow-up. The curves tend to split even within the first year of observation.

In univariate and multivariate analyses, the rs12526453 SNP of the *PHACTR1* gene was one of the variables associated with 5-year survival. The CC homozygotes had a significantly lower probability of death (OR = 0.64, 95% CI 0.41–0.98, p = 0.04). The results of univariate and multivariate analyses are shown in Table 2.

**Table 2:** A univariate analysis for 5-year mortality in the derivation cohort.

Variable	Odds ratio (95% CI)	P
Univariate analysis		
CC genotype	0.64 (0.41–0.98)	0.04
Age (years)	1.06 (1.04–1.09)	<0.0001
Type 2 diabetes	2.2 (1.4–3.5)	0.0007
Smoking	0.63 (0.3–1.3)	0.23
Arterial hypertension	1.8 (1.2–2.85)	0.007
Systolic blood pressure (mmHg)	0.991 (0.98–0.998)	0.02
Heart rate (beats/min)	1.017 (1.005–1.03)	0.0045
Killip class	2.0 (1.6–2.5)	<0.0001
Body mass index (kg/m2)	0.99 (0.89–1.1)	0.95
Creatinine (mg/dl)	2.3 (1.4–3.8)	0.0007
Total cholesterol (mg/dl)	0.99 (0.98–0.99)	0.0002
LDL cholesterol (mg/dl)	0.99 (0.98–0.99)	0.005
Ejection fraction (%)	0.94 (0.92–0.96)	<0.0001
TIMI 3 flow after invasive procedure	0.39 (0.21–0.73)	0.003
Number of vessels with >70% stenosis	1.4 (1.1–1.8)	0.007
Grace risk score	1.02 (1.016–1.03)	<0.0001
Multivariate analysis	Chi^2^ = 81.74	
CC genotype	0.56 (0.35–0.9)	0.016
Age (years)	1.06 (1.03–1.08)	<0.0001
Killip class	1.6 (1.2–2.1)	0.0004
Ejection fraction (%)	0.96 (0.94–0.98)	0.002

For all parameters only admission values were used.

### Validation group

We validated our results in a group of 348 subjects (detailed baseline characteristics shown in Table 1). These patients had significantly higher mean heart rate, lower mean blood pressure, and more stent implantation procedures compared to the derivation group. The validation group included 50.9% CC homozygotes (n = 177), 39.1% heterozygotes (n = 136) and 10% GG homozygotes (n = 35). No significant deviation from the Hardy-Weinberg equilibrium was found (p = 0.25, Chi^2^ test). Differences in genotype distribution between derivation and validation sample were not significant (p = 0.21, Chi^2^ test).

At the cut-off point of 1095 days (3 years), 47 patients had died (13.5%). We confirmed the better prognosis for CC homozygotes in Cox`s F test (p = 0.031). 10.1% of CC homozygotes (n = 18) and 16.95% of G-allele carriers (n = 29), (p = 0.064, Chi^2^ test) died. Kaplan-Meier survival curves for a validation group and specific genotypes are shown in Fig 1B. The univariate and multivariate analyses for validation group and 3-year outcome are shown in Table 3. The CC homozygotes had a trend for better survival (OR = 0.55, 95% CI 0.29–1.04, p = 0.066).

**Table 3:** A univariate analysis for 3-year mortality in the validation cohort.

Variable	Odds ratio (95% CI)	P
Univariate analysis		
CC genotype	0.55 (0.29–1.04)	0.066
Age (years)	1.09 (1.05–1.12)	<0.0001
Type 2 diabetes	1.4 (0.7–2.8)	0.39
Smoking	0.59 (0.3–1.2)	0.12
Arterial hypertension	1.5 (0.7–2.9)	0.26
Systolic blood pressure (mmHg)	1.0 (0.98–1.01)	0.92
Heart rate (beats/min)	1.012 (0.99–1.03)	0.18
Killip class	1.86 (1.3–2.8)	<0.0017
Creatinine (mg/dl)	8.3 (3.2–21)	<0.0001
Total cholesterol (mg/dl)	0.992 (0.98–0.999)	0.037
LDL cholesterol (mg/dl)	0.99 (0.98–1.0)	0.07
Ejection fraction (%)	0.93 (0.89–0.96)	<0.0001
TIMI 3 flow after invasive procedure	1.64 (0.5–5.6)	0.42
Number of vessels with >70% stenosis	1.46 (0.98–2.1)	0.06
Grace risk score	1.02 (1.01–1.03)	<0.0001
Multivariate analysis	Chi^2^ = 63.1	
Age (years)	1.1 (1.05–1.14)	<0.0001
Creatinine (mg/dl)	4.7 (1.6–13.4)	0.004
Ejection fraction (%)	0.92 (0.88–0.96)	<0.0001

For all parameters only admission values were used.

We found no association between rs12526453 genotype and heart rate on admission in this group (79.7 ± 16.3 years in CC homozygotes vs. 79.8 ± 18.15 years in G-allele carriers, p = 0.64, Mann-Whitney test). The other clinical parameters were also comparable between the genotypes.

### Microarray analysis

**Fig 2:**
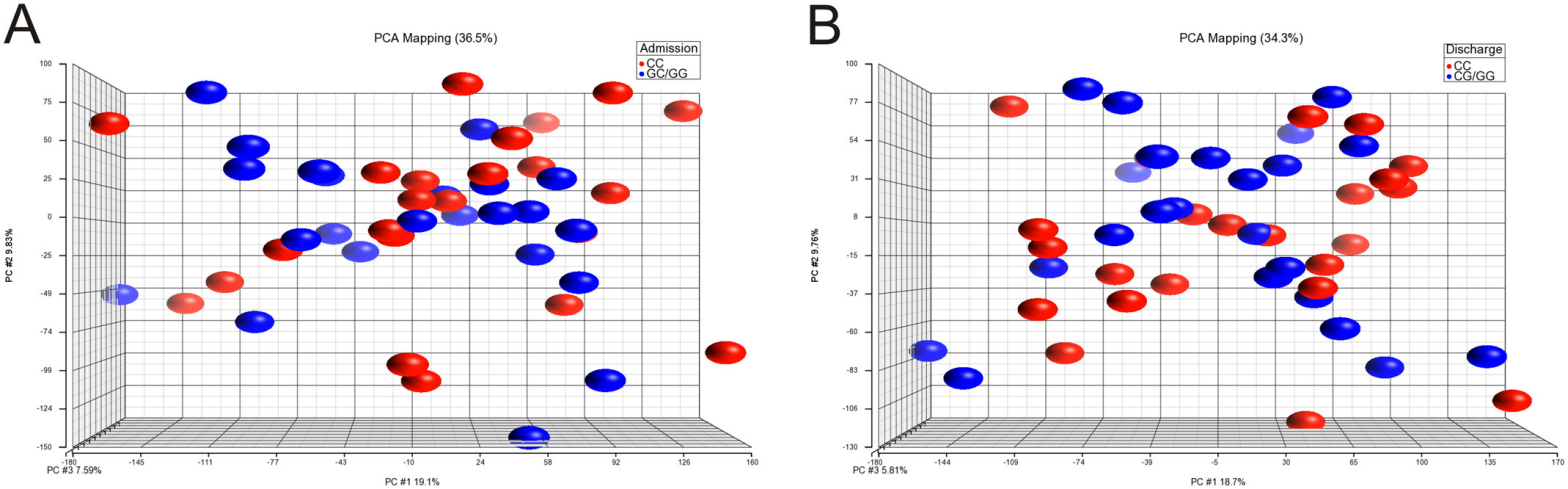
Principal component analysis of PBMCs gene expression profiles. PCA plot shows the first three principal components of microarray data in respect to their correlation.

The microarray data after normalization were subjected to PCA analysis in order to visualize gene expression differences between patients according to the *PHACTR1* polymorphism. Neither the PCA plot (Fig 2) nor the heat map presenting hierarchical clustering (S1 Fig) demonstrate apparent separation between CC and CG/GG genotypes on the 1st day of myocardial infarction (Fig 2A) and after 4–6 days (Fig 2B).

Transcriptomic analysis of the samples from patients on admission (0–24 h) showed the altered expression of 31 transcripts. Only 7 of these were annotated (S2 Table). Consequently, analysis of the samples from the same patients on discharge displayed 44 genes with differentially expressed transcripts. Fourteen of these were annotated (S2 Table). Network analysis of the array results is presented in S2 File.

**Fig 3:**
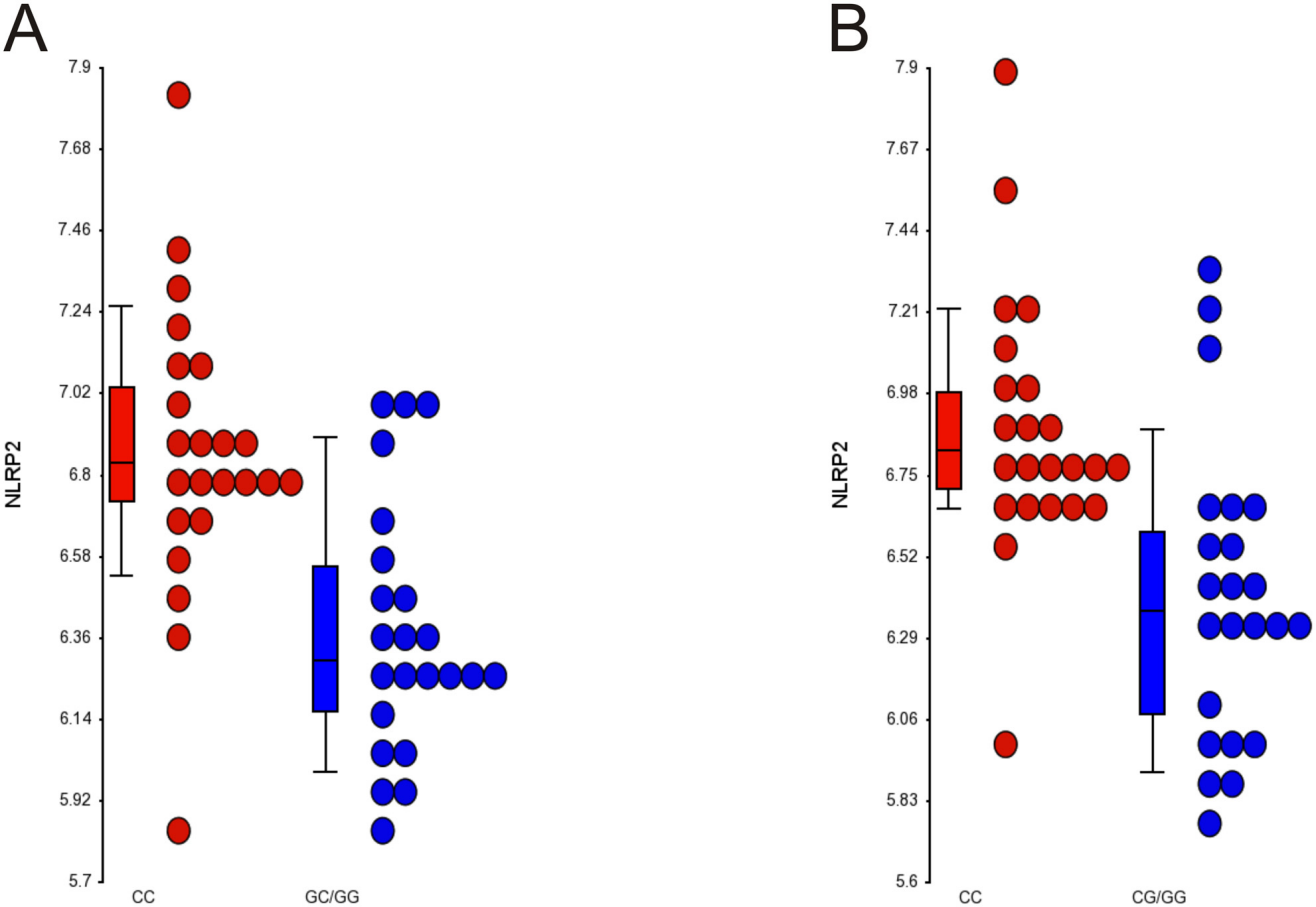
The expression data from microarrays experiments for *NLRP2* gene on admission (A) (p = 0.00001) and discharge (B) (p = 0.0003). The points present the expression level from all analyzed patients with CC genotype (red) and CG/GG (blue).

A comparison of the list of differentially expressed genes at both time points revealed one common transcript–the *NLRP2* gene (a pyrin domain containing 2 [ATPase associated with a variety of cellular activities], GenBank accession number **AK000517)** similarly altered in both analyses (Fig 3A and 3B). Further verification of *NLRP2* expression by means of qPCR confirmed that on the 1^st^ day of myocardial infarction, it is up-regulated in the CC homozygotes group (in comparison to G-allele carrier patients) by a mean factor of 2.795 (S.E. range is 1.293–5.565; p<0.001). These differences remain stable on the 4th-6th days after MI (2.85 fold, S.E. range is 1.424–5.411; p<0.001). Accordingly, there is a significant difference between the homozygotes CC group of patients compared to G-allele carrier patients.

## Discussion

The rs12526453 CC homozygotes are associated with increased risk of coronary artery disease and myocardial infarction [1, 2, 5]. Surprisingly, those potentially high-risk patients showed in 2 independent samples better long-term survival.

### Clinical and genetic paradoxes after myocardial infarction

There are several examples of analogical paradoxes on the level of clinical parameters that increase the risk of coronary events but differentially affect subsequent prognosis—such as obesity, hypercholesterolemia (also observed in our research) and male gender [17, 20]. They tend to be attributed to a favorable clinical profile (younger with less concomitant diseases) and a more aggressive treatment of patients presenting a typical history of risk factors. Despite these explanations, in some reports the mentioned risk factors remain independently associated with a better outcome in multivariate analysis [20]. Furthermore, there is also an example of such a paradox on the genetic level described for the 9p21.3 locus [21–23]. In some studies the 9p21.3 polymorphisms linked with increased risk for coronary artery disease or myocardial infarction were associated with an improved subsequent prognosis. In the INFORM cohort this effect was limited to Caucasians [22].

#### Potential link between *PHACTR1* SNP and survival after STEMI

The mechanism that links *PHACTR1* polymorphism with cardiovascular disease is not sufficiently described. The genotype may play different roles and therefore have opposite effects, depending on clinical settings. CC homozygosity predisposes to coronary artery disease, but in the patients with myocardial infarction from our study, for some reason, it improves survival. We did not find a distinct association of *PHACTR1* polymorphism with most clinical characteristics.

It is worth noting that better survival of high-risk homozygotes after myocardial infarction may influence the results of all studies based on cases and controls comparison, including genome-wide association studies. This could be mainly due to an overrepresentation of myocardial infarction survivors with specific genotypes. Reports by the Myocardial Infarction Genetic Consortium, however, have focused on early-onset myocardial infarction cases (≤ 50 years in males or ≤ 60 years in females), in which case such an effect should be diminished [1].

In general, the link between the rs12526453 SNP and cardiovascular diseases is unclear. This SNP is located in a non-coding region of the *PHACTR1* gene and its influence on the gene function has never been reported. Neither did we find any association of rs12526453 with *PHACTR1* transcript abundance in peripheral blood mononuclear leukocytes. The PHACTR1 is an inhibitor of protein phosphatase 1- a multifunctional enzyme that dephosphorylates serine and threonine residues of numerous substrates and in this way influences various cellular processes. It has been shown to be involved in the regulation of endothelial nitric oxide [24] and to be elevated in patients with end-stage heart failure [25]. Finally, one recent study has proved that it is one of the VEGF effectors that play a role in endothelial cell survival and tube formation [13]. In general, there is still very little data on the subject, and the patomechanism linking this SNP with cardiovascular diseases remains to be elucidated.

In order to analyze the potential mechanisms involved in rs12526453 effects on prognosis after MI, we performed a transcriptomic analysis in the mononuclear blood cells. We found relatively little change in the RNA pattern dependent on the rs12526453 genotype. General association analysis suggests some effect of the CC genotype on inflammatory response, cell-mediated immune response, and cellular development. A striking example of this association here is the augmented expression of the *NLRP2* gene in CC homozygotes with STEMI, both on admission and on discharge. The *NLRP2* gene (also known as Nalp2/Pan1/Pypaf2 **AK000517**) belongs to the nucleotide oligomerization and binding domain (NOD)-like receptor (NLR) family that comprises more than 20 members [26]. It has been shown to modulate an inflammatory response, predominantly by influencing the activity of NF-kB [27]. But there are also reports of other effects of NLRP2, like regulation of early embryonic development [28], or influence on imprinting mechanisms [29]. There are no reports of regulation of NLRP2 by *PHACTR1* and this interaction is yet unknown. In our study we were not able to establish any mechanistic link between these two, either. However, given the established role of NLRP2 as an inflammatory process modulator and its higher expression in CC homozygotes, one may well imagine this phenomenon as the intermediating process in the effect of rs12526453 SNP on long term prognosis after myocardial infarction. Moreover, having in mind the differential effects of NLRP2 on inflammation in different circumstances and tissues [26], it may also theoretically help to explain the paradox that allele C, which increases the risk of MI, provides protection once MI has occurred. In our population, however, the C-reactive protein, the most popular inflammatory response parameter, was not different between CC and GC/GG patients (data not shown).

We did not confirm an association between a prevalence of rs12526453 SNP and myocardial infarction. Nevertheless the study was not designed and not powered sufficiently to investigate this phenomenon that has already been proved in several genome-wide association studies on substantially larger sample sizes. Previously reported odds ratios for association between the C allele and coronary artery disease were relatively low: 1.1–1.15 [1, 4].

This is the first such study that shows better prognosis after myocardial infarction in the case of CC homozygotes. Furthermore, it confirms this phenomenon in an independent sample of patients. Due to the relatively high effect size (odds ratio 0.63) rs12526453 genotyping could be a potentially useful tool for everyday clinical practice to stratify patients after acute coronary syndromes and to tailor appropriately aggressive therapy. Moreover, given the presented data there might be more research necessary to analyze the biological nature of this phenomenon.

### Study limitations

Like in every genetic association study, various confounders may have occurred and influenced our results. The rs12526453 genotype may be just a marker of other conditions associated with outcome, especially comorbidities. We were able to verify only basic such conditions and found a significantly lower heart rate on admission in CC homozygotes in the derivation group. This was not confirmed in the validation group and was probably an accidental finding. CC homozygotes also tended to have lower ejection fraction, more frequently successful angioplasty, and anterior MI. Nevertheless, general risk assessment with the Grace risk score showed comparable risk profile in both genotypes. After adjusting for available conditions, the rs12526453 genotype remained a variable significantly associated with outcome in the derivation group. There are still several confounders that we were unable to adjust for: our database lacks treatment after discharge, time from onset of symptoms to intervention and, in the case of the validation group, also BMI. Next, smoking status was missing in substantial number of patients from the derivation group. We did not verify confounders associated with genetic background. Due to high genetic homogeneity in our population (all patients of European descent, low migration in the region) we may expect relatively high relatedness between the subjects. We were unable to assess it either from pedigrees, or from SNPs estimation (which is possible only in GWAs).

The associations between several variables and outcome were not replicated in the validation group (*PHACTR1* genotype, type 2 diabetes, systolic blood pressure and heart rate on admission, TIMI flow grade after procedure). This might be due to lower number of patients and events as well as differences in clinical characteristics between the 2 populations (Table 1). We aimed to include in univariate and multivariate analyses all types of variables, therefore we chose the logistic regression as a tool. Nevertheless, in the case of the *PHACTR1* genotype, Kaplan-Meier method with Cox`s F test is more sensitive, because it takes into account not only outcome, but also time-to-event, which is especially important in such long-term follow-up.

The other limitations of the study include a relatively small number of patients was enrolled. Nevertheless, we aimed to search for an association of strong effect size and real clinical importance. Very large sample sizes are sufficient to prove significant associations even for extremely small effects. And such a finding may never play a role in everyday clinical practice. Next, patients from the validation group had only a 3-year follow-up performed (in contrast to the 5-year one in the derivation group). Still, we find it sufficient for our analysis, as the differences between the genotypes formed relatively early during observation. Furthermore, our working hypothesis was that CC homozygosity had already been associated with an adverse outcome. The results were opposite to expectations and so far there is no clear explanation for this phenomenon. Finally, our research does investigate the mechanism of how s12526453 affects *NLRP2* expression and survival after MI.

### Conclusions

The rs12526453 single nucleotide polymorphism of the *PHACTR1* gene is associated with 5-year mortality in patients with STEMI. The finding of such a high effect size, after appropriate validation, could potentially be translated into clinical practice. CC homozygotes present a higher expression of the *NLRP2* gene in leukocytes after myocardial infarction; therefore, this gene might play a role in the effects of rs12526453 on prognosis after myocardial infarction.

## Supporting Information

S1 Fig Heat maps presenting hierarchical clustering of transcripts analyzed in microarrays in relation to PHACTR-1 haplotype. Panel A presents results obtained from samples collected after admission. Panel B presents data from samples drawn at discharge.
(TIF)

S2 Fig IPA generated network of differentially expressed genes on admission (A) and at discharge (B). (TIF)

S1 File Supporting Information—methods. (DOCX)

S2 File Supporting Information—results. (DOCX)

S1 Table General characteristics of the genomic substudy group. Data are presented as mean (standard deviation) or n (%) when applicable.
* p = 0.049 for comparisons between CC and GC or GG group.
** 24–72 hours from admission.
(DOC)

S2 Table Differentially expressed genes: CC versus CG/GG. (DOC)

S3 Table Details of primers used in quantitative PCR. (DOC)
