# Supplementary figures and images for: The rs12526453 Polymorphism in an Intron of the PHACTR1 Gene and Its Association with 5-Year Mortality of Patients with Myocardial Infarction
Source: PLoS One. 2015 Jun 18;10(6):e0129820. doi: 10.1371/journal.pone.0129820 (PMC4472810; doi:10.1371/journal.pone.0129820)

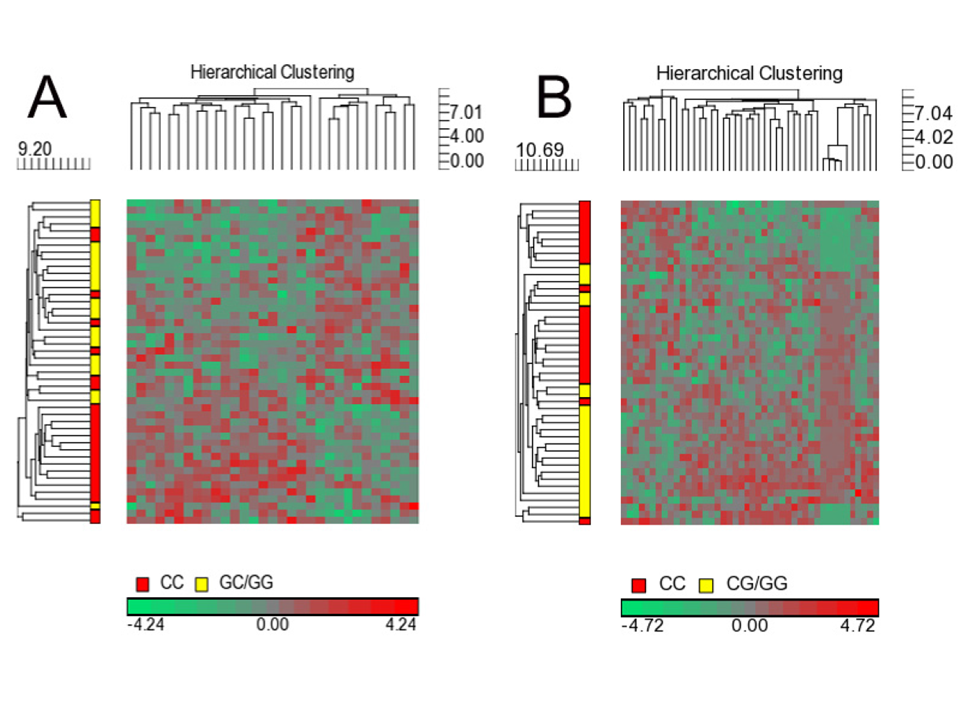

Supplement: S1 Fig — Panel A presents results obtained from samples collected after admission. Panel B presents data from samples drawn at discharge. (TIF) [file pone.0129820.s001.tif]

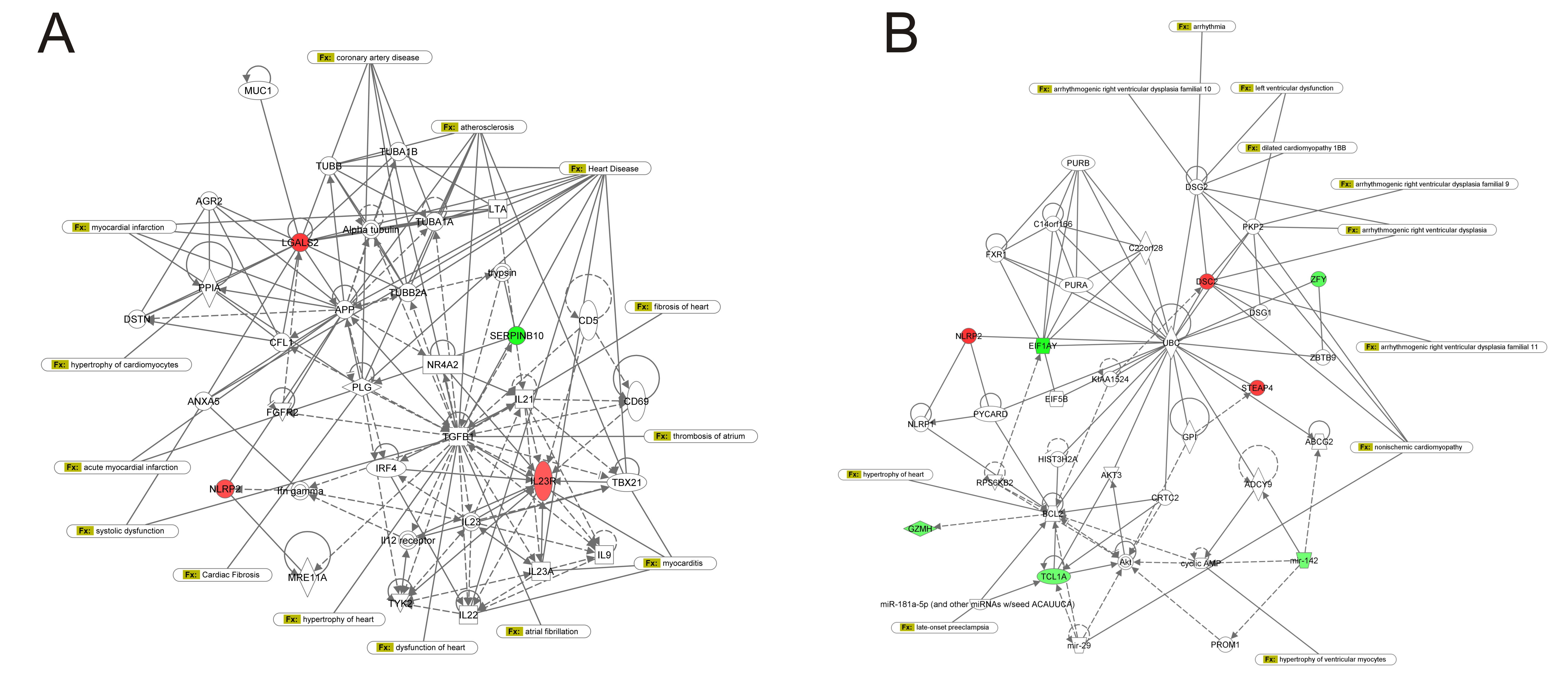

Supplement: S2 Fig — (TIF) [file pone.0129820.s002.tif]
