# Supplementary material for: The rs12526453 Polymorphism in an Intron of the PHACTR1 Gene and Its Association with 5-Year Mortality of Patients with Myocardial Infarction
Source: PLoS One. 2015 Jun 18;10(6):e0129820. doi: 10.1371/journal.pone.0129820 (PMC4472810; doi:10.1371/journal.pone.0129820)
Supplement: S1 File — (DOCX) [file pone.0129820.s003.docx]

**Supporting Methods**:

To compare transcriptomic data of CC patients with GC or GG patients, 46 subjects with STEMI were selected (23 in each group), matched for age. Their clinical characteristics is presented in the S1 Table. Sodium-heparinized blood was collected at the two time points: 0-24 hour from admission and after 4-6 days (at discharge). Peripheral blood mononuclear cells (PBMC) were purified using BD Vacutainer® CPT™ Cell Preparation Tube according to the manufacturer instructions (Becton, Dickinson and Co, Franklin Lakes, NJ,USA). Total RNA was isolated from PBMCs using MagNA Pure Compact System (Roche Diagnostics GmbH, Germany).

GeneChip® HUGene 1.0 ST Arrays (Affymetrix, Santa Clara, CA, USA) was used and processed according to the manufacturer's recommendations. First, the samples were reverse transcribed and amplified, then fragmented and biotinylated. Hybridyzation to the GeneChips® was conducted for 16 h at 45 ˚C. After hybrydyzation the microarrays were washed and stained on a fluidics station and scanned in an Affymetrix GCS 3000 GeneArray Scanner.

After a robust multiarray average (RMA) normalization of microarray data, differences in gene expression levels were calculated by comparing the group with different genotype of PHACTR gene (CC versus CG/GG) at 1st day of myocardial infarction (admission) and after 4-6 days (discharge) independently. Lists of differentially expressed genes and principal component analysis (PCA) were generated using the Partek Genomics Suite software (Partek Inc, St. Louis, MO, USA). The fold change (FC) of gene expression ratios ≥1.3 and P≤0.05 were set as significant criteria. Comparison between group with CC genotype against CG/GG genotype was performed. Lists of genes showing significant differences in expression levels between groups were submitted to Ingenuity Pathway Analysis (Ingenuity® Systems, www.ingenuity.com) and subjected to network analyses.

The real-time reverse transcription-polymerase chain reaction (RT-qPCR) was used to validate the microarray data. Total RNA samples (200 ng) from PBMCs of 26 randomly chosen patients (13 samples of homozygotes CC patients and 13 samples of G-allele carrier patients) was reverse transcribed using QuantiTect Reverse Transcription (Qiagen Gmbh, Germany) according to the manufacturer's recommendations. Primer sets were designed using Clone Manager Suite software (Sci Ed Software, NC, USA); their sequences and reaction conditions parameters are available in S3 Table. qPCR amplification was performed for each sample in triplicate in 96-well plates using LightCycler®480 and LightCycler®480 FastStart SYBR Green I Master (Roche Diagnostics GmbH, Germany). Specificity of the amplified product was confirmed by melting curve analysis and agarose gel electrophoresis (data not shown). Quantification cycles (Cq) were calculated by LightCycler®480 Software, Version 1.5 provided by Roche. The fold change in gene expression levels, corrected by efficiency, was analyzed using REST 2009 version 2.0.13 software (Qiagen Gmbh, Germany) [1]. The expression data were normalized to the two reference genes: hypoxanthine phosphoribosyltransferase 1 (Hprt1) and tubulin, beta class I (Tubb). All experiments (sample collection, RNA preparation and storage, primer design, qPCR normalization) were performed according to the MIQE guidelines [2].
