# Supplementary material for: The rs12526453 Polymorphism in an Intron of the PHACTR1 Gene and Its Association with 5-Year Mortality of Patients with Myocardial Infarction
Source: PLoS One. 2015 Jun 18;10(6):e0129820. doi: 10.1371/journal.pone.0129820 (PMC4472810; doi:10.1371/journal.pone.0129820)
Supplement: S2 File — (DOCX) [file pone.0129820.s004.docx]

**Supporting Results**

Functional analysis of differentially expressed genes between genotypes was undertaken in order to assess the differences in gene expression according to genotype. Both lists of differentially expressed genes in investigated time points associated with genotype were analyzed separately in IPA. According to the top network the differentially expressed genes on admission were connected with Inflammatory Response, Cell-mediated Immune Response and Cellular Development (Supporting Information Fig 2A.). In contrast, on discharge the differentially expressed genes were involved in Cardiovascular Disease, Congenital Heart Anomaly and Developmental Disorder (Supporting Information Fig S2.B).

**Supporting references**

1. Pfaffl MW, Horgan GW, Dempfle L. Relative expression software tool (REST) for group-wise comparison and statistical analysis of relative expression results in realtime PCR. Nucleic Acids Res 2002;30: e36.
2. Taylor S, Wakem M, Dijkman G, Alsarraj M, Nguyen M. A practical approach to RTqPCR- Publishing data that conform to the MIQE guidelines. Methods 2010;50: S1-5.
