# Supplementary material for: The rs12526453 Polymorphism in an Intron of the PHACTR1 Gene and Its Association with 5-Year Mortality of Patients with Myocardial Infarction
Source: PLoS One. 2015 Jun 18;10(6):e0129820. doi: 10.1371/journal.pone.0129820 (PMC4472810; doi:10.1371/journal.pone.0129820)
Supplement: S1 Table — Data are presented as mean (standard deviation) or n (%) when applicable. * p = 0.049 for comparisons between CC and GC or GG group. ** 24–72 hours from admission. (DOC) [file pone.0129820.s005.doc]

**S1 Table. General characteristics of the genomic substudy group.** Data are presented as mean (standard deviation) or n (%) when applicable.

|  | Total (n=46) | CC (n=23) | GC or GG (n=23) |
| --- | --- | --- | --- |
| Age | 56.7 (10.2) | 58.0 (9.6) | 55.5 (10.7) |
| Women* | 12 (26%) | 9 (39%) | 3 (13%) |
| Hypertension | 19 (41%) | 10 (43%) | 9 (39%) |
| Diabetes | 9 (20%) | 5 (22%) | 4 (17%) |
| Previous myocardial infarction | 1 (2.1%) | 0 (0%) | 1 (4.3%) |
| Creatinine (mg/dl) | 0.92 (0.16) | 0.87 (0.14) | 0.97 (0.17) |
| STEMI anterior | 18 (39%) | 8 (35%) | 10 (43%) |
| EF** | 49.4 (8.4) | 50.2 (7.3) | 48.5 (9.4) |

* p=0.049 for comparisons between CC and GC or GG group

** 24-72 hours from admission
