# Supplementary material for: The rs12526453 Polymorphism in an Intron of the PHACTR1 Gene and Its Association with 5-Year Mortality of Patients with Myocardial Infarction
Source: PLoS One. 2015 Jun 18;10(6):e0129820. doi: 10.1371/journal.pone.0129820 (PMC4472810; doi:10.1371/journal.pone.0129820)
Supplement: S2 Table — (DOC) [file pone.0129820.s006.doc]

**S2 Table**. Differentially expressed genes: CC versus CG/GG

| **Gene Symbol** | **GenBank Accession Number** | **Gene ID** | **RefSeq** | **Entrez Gene Name** | **Fold Change** | **p-value** |
| --- | --- | --- | --- | --- | --- | --- |
| **Differentially expressed genes at admission** | | | | | | |
| **NLRP2** | **AK000517** | 55655 | NM_017852 | NLR family, pyrin domain containing 2 | 1.39949 | 0.0001 |
| **RNU4-2** | **K00473** | 26834 | NR_003137 | RNA, U4 small nuclear 2 | 1.60638 | 0.0014 |
| **IL23R** | **AF461422** | 149233 | NM_144701 | interleukin 23 receptor | 1.30145 | 0.0035 |
| **LGALS2** | **BC059782** | 3957 | NM_006498 | lectin, galactoside-binding | 1.55321 | 0.0159 |
| **RNU5E** | **M77839** | 26829 | NR_002754 | RNA, U5E small nuclear | 1.30213 | 0.0325 |
| **FAM154B** | **AL833762** | 283726 | AK304339 | family with sequence similarity 154, member B | 1.35366 | 0.0454 |
| **SERPINB10** | **U35459** | 5273 | NM_005024 | serpin peptidase inhibitor, clade B (ovalbumin), member 10 | -1.34399 | 0.0495 |
| **Differentially expressed genes on discharge** | | | | | | |
| **NLRP2** | **AK000517** | 55655 | NM_017852 | NLR family, pyrin domain containing 2 | 1.39165 | 0.0003 |
| **MIR142** | **AC004687** | 406934 | NR_029683 | microRNA 142 | -1.31737 | 0.0006 |
| **GZMH** | **BC027974** | 2999 | NM_033423 | granzyme H (cathepsin G-like 2, protein h-CCPX) | -1.418 | 0.0013 |
| **TCL1A** | **BC014024** | 8115 | NM_021966 | T-cell leukemia/lymphoma 1A | -1.37681 | 0.0112 |
| **DSC2** | **BC063291** | 1824 | NM_024422 | desmocollin 2 | 1.31869 | 0.0147 |
| **STEAP4** | **DC333286** | 79689 | NM_024636 | STEAP family member 4 | 1.3149 | 0.0164 |
| **CCDC23** | **BQ712089** | 374969 | NM_199342 | coiled-coil domain containing 23 | -1.39076 | 0.0206 |
| **IGLV7-46** | **Z73674** | 28775 | ENST00000390295 | immunoglobulin lambda variable 7-46 (gene/pseudogene) | -1.44672 | 0.0215 |
| **EIF1AY** | **AF000987** | 9086 | NM_004681 | eukaryotic translation initiation factor 1A, Y-linked | -2.10257 | 0.0371 |
| **OR2L8** | **BK004459** | 391190 | NM_001001963 | olfactory receptor, family 2, subfamily L, member 8 | 1.30083 | 0.0382 |
| **LOC162632** | **BC039345** | 162632 | NR_003190 | TL132 pseudogene | -1.36825 | 0.0458 |
| **PRKY** | **BC074852** | 5616 | NR_028062 | protein kinase, Y-linked | -1.47703 | 0.0459 |
| **ZFY** | **L10393** | 7544 | NM_003411 | zinc finger protein, Y-linked | -1.54268 | 0.0476 |
