# Supplementary material for: The rs12526453 Polymorphism in an Intron of the PHACTR1 Gene and Its Association with 5-Year Mortality of Patients with Myocardial Infarction
Source: PLoS One. 2015 Jun 18;10(6):e0129820. doi: 10.1371/journal.pone.0129820 (PMC4472810; doi:10.1371/journal.pone.0129820)
Supplement: S3 Table — (DOC) [file pone.0129820.s007.doc]

**S3 Table**. Details of primers used in quantitative PCR

| **Gene symbol** | **Gene name** | **GenBank ID**  **for mRNA record** | **Sequence of primer** | **Amplicon size (bp)** | **Efficiency** | **Tm (˚C)** | **Extension time** |
| --- | --- | --- | --- | --- | --- | --- | --- |
| *Hprt1* | hypoxanthine phosphoribosyltransferase 1 | NM_000194 | F: TGACCTTGATTTATTTTGCATACC  R: CGAGCAAGACGTTCAGTCCT | 102 | 0,74 | 55 | 5‘‘ |
| *Tubb* | tubulin, beta class I | NM_178014 | F: CTTCAAGCGCATCTCGGAGC  R: TGCGGTGGCATCCTGGTACT | 119 | 0,91 | 61 | 5‘‘ |
| *Nlrp2* | NLR family, pyrin domain containing 2, transcript variant 1 | NM_017852 | F: TTGGCCTCGCTAACGAGAAG  R: GCAGGTCTGTCACCGTTGAA | 135 | 0,79 | 53 | 7‘‘ |
